# Factors affecting households’ trust in the community based health insurance scheme in Ethiopia

**DOI:** 10.1371/journal.pgph.0000375

**Published:** 2022-05-04

**Authors:** Wakuma Akafu Eseta, Shimeles Ololo Sinkie

**Affiliations:** Department of Health Policy and Management, Faculty of Public Health, Institute of Health, Jimma University, Oromia, Ethiopia; University College London, UNITED KINGDOM

## Abstract

The trust that potentially community-based health insurance (CBHI) members have in the actual health insurance scheme is a crucial determinant for members who decided to enroll and continue being members of the scheme. While the literature on health insurance in low-income countries mentions the importance of trust in consumers’ decision to insure, to date the association between trust and trust-building factors has not been researched in the Ethiopian context. Therefore, the objective of this study was to assess the factors affecting households’ trust in the CBHI scheme in Ethiopia. A community-based cross-sectional study design was employed to collect data from 617 household heads from March 1–30, 2020. A multistage sampling technique was carried out and interviewer-administered questionnaires were used to collect data. Descriptive statistics and multivariable linear regression analyses were performed, and variables with a *P*-value < 0.05 were considered to have a statistically significant association with trust in the CBHI scheme. A household survey resulted in 617 households being interviewed making a response rate of 97.3%. The mean age of the respondents was 44.7 (SD°11.2) years and the majority of the respondents were Oromo 528(85.6%). Holding other covariant fixed, educational level of household head (ß = 0.17, 95% CI:0.14–0.32), members’ satisfaction with past experience (ß = 0.40, 95%CI:0.41–0.57), favorable attitude toward CBHI (ß = 0.31, 95%CI:0.12–0.49), perceived quality of service (ß = 0.15, 95% CI:0.02–0.29) and dropout from CBHI (ß = -0.23, 95%CI:- 0.41–- 0.05) were covariant that had an association with trust in the CBHI scheme. This study found that the educational level of the household head, members’ satisfaction with past experience, members’ attitude toward CBHI, perceived quality of services and dropout from CBHI were covariant that were affecting trust in the CBHI scheme. Therefore, if the scheme wants to build trust and maintain membership, great attention should be given toward the improvement of the quality of service and attitude of members towards the CBHI scheme. These findings suggest that CBHI managers, different health insurance agencies, providers and policymakers need to think systematically about a wide range of initiatives that enhance trust and caring, and to design trust-building structures and practices that sustain the CBHI scheme.

## Introduction

Community Based health insurance (CBHI) is one of health care financing reform and is defined as the reduction or elimination of uncertain risk of loss for individuals or households by combining a large number of similarly exposed individuals or households who are included in a common fund that makes good the loss caused to any one [1]. In health insurance schemes, people who are at a similar risk of a certain event contribute a small amount as a premium to a health insurance fund to be used to treat people who become ill. They are designed to be simple and affordable and to draw on resources of social solidarity and cohesion to overcome problems of small risk pools, moral hazard, fraud, exclusion and cost-escalation [2]. It is also called Community health insurance, Community health financing, community prepayment, micro health insurance and mutual health organizations [3, 4].

CBHI is characterized by the following: the community is involved in driving its setup and management; It is a prepayment mechanism with pooling of health risks and funds taking place at the level of the community or a group of people, Premiums are often a flat rate and independent of individual health risks; Entitlement to benefits is linked to making a contribution; Affiliation is voluntary, and Operate on a non-profit basis [5]. The CBHI targets informal sector employees of urban and rural residents [6, 7].

In fact, there is strong evidence that CBHI enhances resource mobilization to improve health service utilization, quality of life, reduce out-of-pocket (OOP) payments and reduce reliance on potentially harmful coping responses such as borrowing or selling assets [8–12]. These systems also intend to respond to the goal of fairness in the financing, in that beneficiaries are asked to pay according to their ability while guaranteeing them the right to health services according to their needs [13].

As a part of its strategy to achieve universal health coverage, the government of Ethiopia piloted the CBHI in 13 pilot districts in 2011. Three years on, it was decided to scale up it in 161 districts. The evaluation of the impact of the pilot schemes was made which is intended to inform the scale-up process and the government has been focusing on expanding it since 2015. This program aimed to cover citizens in the rural and informal sectors estimated to be 85% of the country’s population, which calls for 80% of the districts enrolling at least 80% of eligible households by 2020 [14]. However, national overall enrollment is very low which was 48% and 36% in 2013 and 2017 respectively [8, 15] and dropout from the scheme is increasing from time to time despite of the different advantages of the CBHI [16, 17].

As cited by Ozewa and Walker, Dubois et al showed that the main characteristic that differentiates CBHI from other health insurance like social health insurance is that enrollment is voluntary which gives credence to the hypothesis that trust in health insurers may matter when people decide whether to enroll as well as continue being members of the CBHI scheme. In addition, health insurance is based on the fundamental idea that there is a level of uncertainty about future health outcomes, and that this risk could be transferred to another party in advance. Prepayment is made to transfer this risk to the insurer, in exchange for an agreement that the insurer will reimburse the insurance for covered losses in the future. Thus, trust may play a critical role in the local demand for the CBHI scheme [18].

Although numerous definitions of trust have been proposed in the medical context scholars share common themes and the majority stress the optimistic acceptance of a vulnerable situation in which the truster believes the trustee will care for the truster’s interests [18, 19]. Trust is inseparable from vulnerability, in that there is no need for trust in the absence of vulnerability. The greater the risk, the greater the potential for either trust or distrust. Trust is sometimes said to create vulnerability, as in an intimate relationship, but vulnerability is primary and unavoidable in public health, and so it is appropriate to think of trust arising from conditions of vulnerability. Considering the profound vulnerability created by illness and invasive treatment, trust in the CBHI scheme can have remarkable strength or resilience [19].

Recent work has begun to highlight the potential value of trust in understanding the performance of health care organizations and public health reform like CBHI as people’s trust in CBHI management was reported to be a facilitator of insurance enrollment and sustainability decisions [20–22]. A study conducted in West Africa found that providers’ inferior quality of care negatively affected membership in the CBHI scheme [4]. In addition, household survey findings from many countries consistently indicate that members are worried about whether their payments to organizations will be used for their benefit [21, 23]. Furthermore, on top of low enrollment, a dropout from the CBHI scheme is the main challenging issue in the sustainability of the scheme in sub-Saharan Africa including Ethiopia [24–27].

Consequently, the CBHI scheme including which manages premium funds as well as providers contracting with CBHI would need to have consumers’ trust before they would be enrolled and contract renewal. Researchers have examined consumer trust in managed care settings [28], popular trust in health systems [29, 30], and patient trust in providers [31, 32]. Yet whilst the literature on health insurance in low-income countries mentions the importance of trust in consumers’ decision to insure and sustain membership [33], to date the association between trust and trust-building factors has not been researched in the Ethiopian context.

Therefore, this study is probably the first in Ethiopia to identify factors affecting trust in health insurance members in the context of the CBHI scheme. A clear understanding of these factors could help policy-makers, managers, health workers, health insurance agencies, and different non-governmental organizations in developing structures and practices that build and sustain trust in the CBHI scheme.

## Methods and materials

### Ethics statement

The ethical clearance was obtained from the ethical review committee of Jimma University, Institute of Health Science (reference number: IRB00052/2020). The necessary permission was obtained from the Manna district health and administrative offices after a formal letter was written from Jimma zonal health department to the district. This study was conducted in accordance with the Declaration of Helsinki. All study participants were well informed about the purpose of the study and informed written consent was secured from the study participants prior to the interview. The study participants’ confidentiality was maintained and no personal identifiers were used in the data collection tools and codes were used in place of it. All paper-based and computer-based data were kept in protected and safe locations. The recorded data were not accessed by a third person, except the research team, and data sharing will be enacted based on the ethical and legal rules of data sharing.

### Study setting and period

The study was conducted from March 1–30, 2020 in the Manna district. The Manna district is one of the 21 districts in the Jimma Zone. It is located 382 km from Addis Ababa, the capital city of Ethiopia, and 32 km away from Jimma town to the west. It is bordered on the South by Seka Chekorsa, on the West by Gomma, on the North by Limmu Kosa, and on the East by Kersa district. The town administrative center is Yebu. It is among the 17 districts in the Jimma zone where the CBHI scheme is currently being implemented. Manna district has a total of 26 Kebeles, 1 urban and 25 rural Kebeles. According to a report obtained from the Manna district health office, the total population of the Manna district was 205497 and among this population, the number of households was estimated to be 42812. The number of members ever registered to the CBHI scheme in the districts since the onset of CBHI was 10713. The district has 7 health centers and 26 health posts.

### Study design and population

A community-based cross-sectional stud was employed. All households who were ever registered to the CBHI scheme in the Manna district were the source population, whereas all households who were registered to the CBHI scheme and found in selected villages of the district that fulfilled the inclusion criteria were the study population and were included in the study.

### Sample size determination

The sample size was determined using a single population proportion formula considering the following assumptions [34]:

$$n=\frac{P_{o}(1‐P_{o}){\left(Z_{\alpha /2}\right)}^{2}}{d^{2}}*D$$

Where

○ n = sample size
○ Z_α/2_ = Standard score for 95% confidence level (1.96)
○ P_o_ = 50% proportion of trust was taken because there is no study conducted in Ethiopia that addresses the objective of this study.
○ d = 5% margin of error
○ D = a design effect of 1.5 was considered because of the two-level random selection of kebeles and participants
○ 10% non-respondents rate was considered

Thus, the final sample size become **634**

### Sampling procedure

A multistage sampling technique was employed to reach study participants. First, the 26 kebeles of the district were stratified into urban and rural kebeles. Then, the urban kebele was purposively selected for representation. Eight out of 25 rural kebeles were selected using the lottery method simple random sampling technique. Then after, the calculated sample size was proportionally allocated to each of the nine selected kebeles based on the number of enrolled households. Finally, the study participants were selected using a computer-generated simple random sampling technique. The lists of household heads that were registered to the CBHI were taken from the district CBHI scheme which was used as a sampling frame. Their usual place of residence was identified in collaboration with Kebele leaders and the head of 1-to-5 networks.

### Data collection tool

An interviewer-administered structured questionnaire was adapted from the related literature to collect relevant information. The questionnaire had five parts: socio-economic and demographic characteristics, CBHI information, individual/household-related factors, CBHI related factors, and health service use related factors. The questionnaire was translated to the local language Afan Oromo by language experts and translated back to English by another person to ensure its consistency and was finally pre-tested before the actual data collection. The internal consistency of items for both independent variables with a five-point Likert scale and dependent variables was checked using Cronbach’s alpha (S1 Text).

### Survey administration

Data were collected by six diploma nurses using a standardized, pre-tested and structured questionnaire through face-to-face interviews. Two supervisors who were qualified with BSc in public health were recruited. Data collectors and supervisors were recruited based on their previous experiences in data collection and fluency in the local language. In addition to the two recruited supervisors, the principal investigator was also closely supervised the data collection process. The data collectors and supervisors were trained for one day on the data collection tool, approach to the interviewees, details of interviewing techniques, respect and maintaining privacy and confidentiality of the respondents.

### Study variables

Trust in the CBHI scheme was a dependent variable, whereas socio-economic and demographic factors (household head’s age, sex, educational status and wealth index), individual/ household related factors (understanding of CBHI, attitude toward CBHI, self-rated health status, perceived quality of service and trust in a health facility), health service use related factors (accessibility of service, availability of service, waiting time and providers’ attitude), and CBHI related factors (affordability, convenience of premium collection period and scheme experience) were independent variables for the study.

### Measurement

#### Trust in the CBHI scheme (dependent variable)

Participants were asked five items with a 5 point Likert scale strongly disagree to strongly agree; then the factor score was computed using principal components analysis (PCA) and used as a continuous variable during the analysis. The Kaiser-Meyer-Olkin (KMO) measure of sample adequacy was 0.66 with significant Bartlett’s test of sphericity. Finally, one component was extracted at one eigenvalue with a total variance of 60.3% and was used as a dependent variable. Internal consistency of the items was checked using Cronbach’s alpha and was found to be 71.1%.

#### Trust in the health care facilities

Participants were asked ten items with a 5 point Likert scale strongly disagree to strongly agree then the factor score was computed using PCA and the score was used as a continuous variable during the analysis. KMO measure of sample adequacy was 0.5 with significant Bartlett’s test of sphericity. Finally, one component was extracted at one eigenvalue with a total variance of 69.5%. The internal consistency of the constructs was checked using Cronbach’s alpha computation and was found to be 86.9%.

#### Provider’s attitude

Participants were asked ten items of question which were measured using 5 points Likert scale ranging from strongly disagree to strongly agree. The factor score was computed using PCA and the extracted factor score was ranked based on percentile then categorized into three dummy variables for analysis whether a member perceives that the provider has an unfavorable, neutral or favorable attitude toward the CBHI member. KMO measure of sample adequacy was 0.67 with significant Bartlett’s test of sphericity. Finally, one component was extracted at one eigenvalue with a total variance of 62.7%. The internal consistency of the items was checked using Cronbach’s alpha and was found to be 86.3%.

#### Scheme experience

Composite variable constructed using seven items measuring the satisfaction of the respondent on the CBHI experience and design feature of the schemes with 5 points Likert scale strongly disagree to strongly agree then, the factor score was computed using PCA and used as a continuous variable during the analysis. KMO measure of sample adequacy was 0.77 with significant Bartlett’s test of sphericity. Finally, one component was extracted at 1 eigenvalue with a total variance of 60.8%. The internal consistency of the items was checked using Cronbach’s alpha and was found to be 85.8%.

#### Perceived quality of health service

This variable was measured on a five-point Likert scale ranging from very poor to very good. Later, the data were regrouped into three categories a poor, medium and good for numerical significance.

#### Attitude toward the CBHI scheme

Participants asked a set of questions containing 10 items that were measured using 5 point liker scale ranging from strongly agree to strongly disagree. Assumption of summated scales was employed to examine the overall score representing the respondent’s position on the continuum of favorableness toward CBHI [34]. Accordingly, ten items had a potential minimum sum score of 10 to a maximum sum score of 50. When the total score of each individual is close to 50 it shows the most favorable attitude and when the score is close to 10 it shows the most unfavorable attitude toward the scheme. Thus based on this continuum of favorableness it was categorized into 3 dummy variables unfavorable attitude, who scored between 10 and 29; neutral attitude, who scored 30; and favorable attitude 31–50. The internal consistency of the constructs was checked using Cronbach’s alpha and was found to be 74.8%.

#### Household wealth index

Household assets were collected based on the kinds of consumer goods they own then factor scores were derived using PCA, and the composite score was categorized into five quantiles. The first 20% quantile was classified as the poorest whereas the last 20% quantile was considered as the richest. The KMO measure of sample adequacy was 0.75 with significant Bartlett’s test of sphericity. Finally, six components were extracted at one eigenvalue and the total variance explained by these variables was 66.7%.

#### Kebele

A kebele (Ganda in Afan Oromo) the so called village is the smallest administrative unit of Ethiopia, similar to a word, a neighborhood or a localized and delimited group of people.

### Data quality control

For the effectiveness and quality of data collection, experts assessed whether the data collection tool measures what it was intended to measure and if it was comprehensive enough to collect all the information needed to address the objective of the study. The reliability of the questionnaire was also assessed. Training was given for one day for the data collectors and supervisors. The pre-tested tool was used for data collection and the non-response rate was reduced by repeated contact. Supervisors were thoroughly checked before receiving the filled questionnaire from each data collector and they randomly selected the questionnaire to crosscheck its completeness.

### Data processing and analysis

After checking the completeness and consistency of the data, the collected and coded data were entered, cleaned and checked by Epi data manager version 3.1 and then exported to SPSS version 26 statistical packages for analysis. The frequency distributions of all variables were examined to check for data entry errors. For both dependent and independent variables, principal component extractions with Eigenvalues greater than one and varimax rotation methods were employed for factor analysis. The KMO measure of sample adequacy above 0.5 with significant Bartlett’s test of sphericity was used.

Finally, the component extracted with total variance explained more than 60% was used. For the factor extracted, the factors were renamed according to the items contained in the extracted items. Items with insignificant loadings (loading below 0.40) and items with cross-loadings were removed from the analysis. An Eigenvalue greater than one decision rule was used to determine the appropriate number of factors to be extracted. Items with Cronbach’s alpha values greater than 0.7 extracted from each of the scales were used in subsequent analyses. The factor score was computed for the outcome variables and treated as a continuous variable for multiple linear regressions.

Descriptive analyses were used to describe trust in the CBHI scheme and presented in terms of frequencies, percentages, tables and graphs as necessary. Simple linear regression analysis was performed to select candidate variables for multivariable analysis. Then, variables with a p-value < 0.25 in a simple linear regression analysis were considered as candidates for multivariable analysis. After checking all necessary assumptions for multivariable linear regression analysis, backward method multivariable linear regression analysis was performed. Then, 95% confidence intervals and beta coefficients were calculated and used to describe statistically significant variables. A *P-*value < 0.05 was considered as a statistically significant determinant of trust of members in the CBHI scheme (S1 Data).

## Results

### Socio-economic and demographic characteristics

A total of 617 households were included in the study making a response rate of 97.3%. The mean age of the respondents was 44.7 (SD ±11.2) years. The majority of the respondents were from rural 544(88.2%) and 522(84.6%) were male. The majority of the respondents were Oromo in ethnicity which accounted for 528(85.6%) and Muslims constitutes 509(82.5%) of the participants. Regarding the educational status of respondents, 313(50.7%) had no formal education. About 451(73%) of the respondents were farmers and the mean family size of the participants was 5.5 (SD±1.9) (Table 1).

**Table 1:** Socio-economic and demographic characteristics of study participants among households in Manna district, Jimma zone, southwest, Ethiopia, 2020.

Variables	Category	Frequency	Percent
Age category in years	18–30	62	10
	31–40	205	33.2
	40–50	197	31.9
	≥51	153	24.8
Residence	Rural	544	88.2
	Urban	73	11.8
Sex	Male	522	84.6
	Female	95	15.4
Marital status	Married	553	89.6
	Divorced	28	4.5
	Separated	18	2.9
	Widowed	18	2.9
Educational level	Unable to read and write	199	32.3
	Able to read and write	114	18.5
	Primary education	181	29.3
	Secondary and above	123	19.9
Household size	≤ 5	301	48.8
	> 5	316	51.2
CBHI status	Renewed	420	68
	Dropped	197	32
Distance from health facility (in Minutes)	< 30	185	30
	≥ 30	432	70
	Yes	366	59.3
Wealth index	Poorest	121	19.6
	Poor	124	20.1
	Middle	125	20.3
	Rich	123	19.9
	Richest	124	20.1

### Community based health Insurance status of study participants

Among the study participants 541(87.7%) were payers. This means that 76(12.3%) of respondents were indigent who were exempted from contributing to CBHI. Almost all of them, 607(98.4%), had CBHI identification cards. Of those who renewed their membership (68%), only 137(32.6%) were renewing consistently over the past five years. Of those who discontinued their membership 103(52.3%) of participants had one years of stay and 7(3.5%) had four year of stay. About 80(19%) and 31(16%) of those who had renewed and dropped from CBHI respectively had no intention to renew their membership in the coming year.

### The attitude of Insured household towards CBHI

According to the assumption of summated scales employed to examine the overall attitude of members towards CBHI, around 87% of households had a favorable attitude toward the CBHI scheme. Surprisingly even among those who discontinued their membership around 77% of households head had a favorable attitude towards CBHI (Table 2).

**Table 2:** Attitude of insured household towards CBHI scheme in Manna district, Jimma zone, southwest, Ethiopia, 2020.

	Strongly disagree		Disagree		Neutral		Agree		Strongly agree	
	No	%	No	%	No	%	No	%	No	%
CBHI has potential in promoting HC seeking Behavior	10	1.6	35	5.7	88	14.3	310	50.2	174	28.2
CBHI protects households from unaffordable health care expense	14	2.3	31	5.0	83	13.5	289	46.8	200	32.4
premium payment for CBHI is expensive	135	21.9	174	28.2	144	23.3	99	16.0	65	10.5
CBHI is a means of collecting revenue(profit) for government	132	21.4	221	35.8	126	20.4	121	19.6	17	2.8
CBHI members receive low quality services than non-members	116	18.8	195	31.6	185	30.0	99	16.0	22	3.6
mistreatment of pt. by the HP is common in members than non-members	155	25.1	195	31.6	158	25.6	88	14.3	21	3.4
I did not have trust in management and administration of CBHI	114	18.5	169	27.4	147	23.8	136	22.0	51	8.3
CBHI is relevant only to promote health condition of the poor	155	25.1	220	35.7	106	17.2	102	16.5	34	5.5
CBHI is good to pool the risk of health expenditure within sick and healthy individual	23	3.7	84	13.6	85	13.8	296	48.0	129	20.9
CBHI should be advocated and scaled up to improve the health of a rural community	21	3.4	36	5.8	73	11.8	241	39.1	246	39.9

### Impacts of CBHI on quality of service (perceived) at contracted health facilities

**Fig 1:**
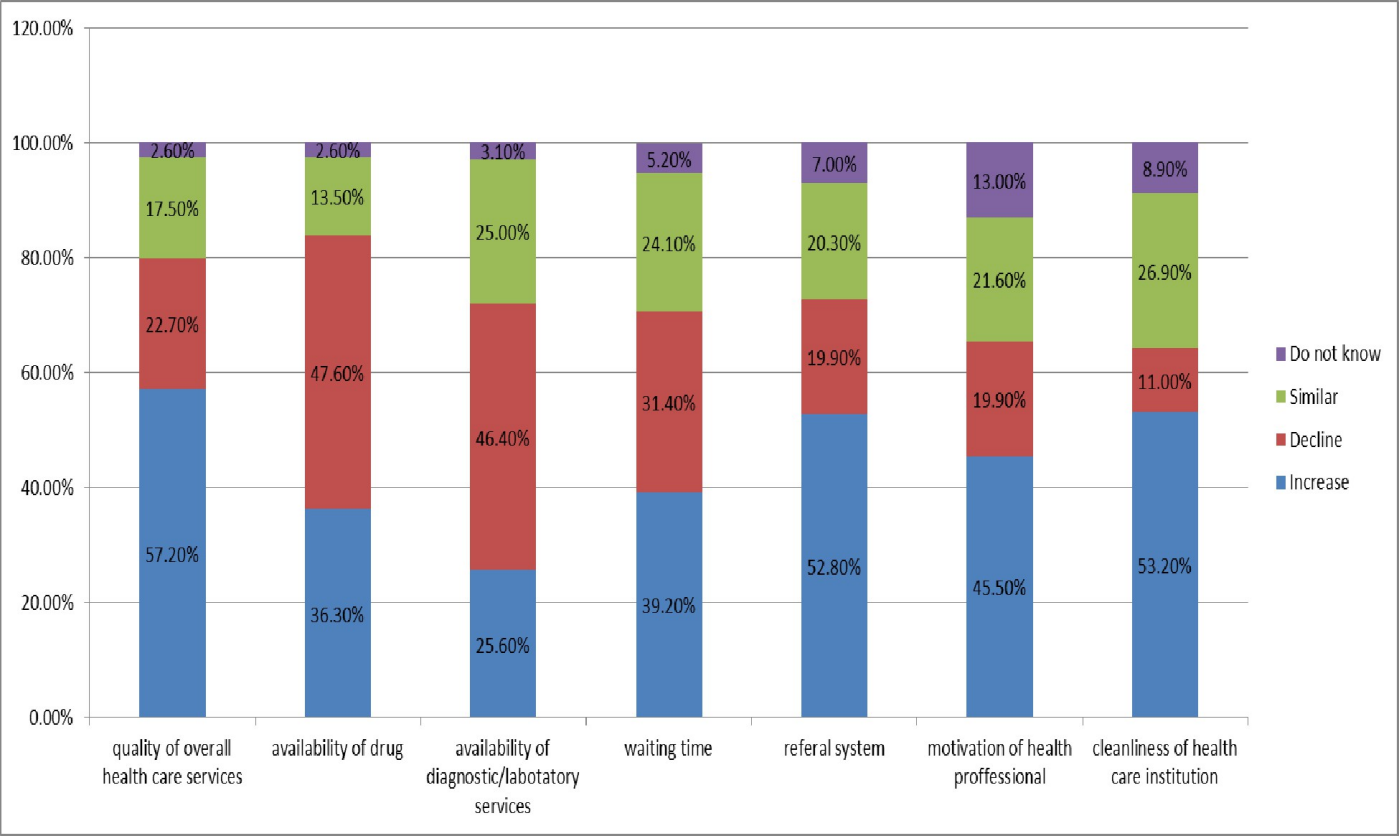
Impacts of CBHI on quality of service (perceived) at contracted health facilities among household in manna District Jimma, South West, Ethiopia, 2020.

As shown in the figure, around 57% of participants perceived that the overall quality of services was increased at contracted health facilities since the onset of community based health insurance. However, around 23% of the study participants perceived that the overall quality of services was decreased at contracted health facilities since the onset of community-based health insurance (Fig 1)

### Simple and multivariable linear regression analysis for factors affecting trust in the CBHI scheme

Candidate variables in simple linear regression analysis were taken to multivariable linear regression analysis to identify possible factors affecting trust in the CBH scheme. The amount of dependent variable explained by independent variable was shown by adjusted R square and it was found to be 44.1%. Multi collinearity between independent variables was checked using variance inflation factor (VIF) and the maximum VIF was found to be 2.014.

This study revealed that households’ educational status has a statistically significant impact on trust in the CBHI scheme. Thus, keeping other variables constant, households with secondary and above educational levels increased their trust score by 0.17 as compared to households who were unable to read and write [ß = 0.17 (95% CI: 0.14–0.32; p = 0.027)] (Table 3).

**Table 3:** Simple and Multivariable linear regression analysis for determinants of trust of member in CBHI scheme among households of Manna district, Jimma zone, southwest, Ethiopia, 2020.

Variables	Univariable linear regression		Multivariable linear regression	
	*B*	95%CI *B*	*B*	95%CI *B*
Household age	0.007	0.00 to 0.014	-0.003	-0.003 to 0.008
Satisfaction of member with experience	0.64	0.578 to 0.69	0.40	**0.41 to 0.57** ^**^
Perceived quality_poor	**Ref**			
Perceived quality_medium	-0.06	0.24 to 0.13	0.06	-0.13 to 0.24
Perceived quality_good	0.73	0.58 to 0.87	0.15	**0.02 to 0.29** ^*^
Educational level_unable to read and write	**Ref**			
Educational level_able to read and write	-0.12	-0.32 to 0.09	0.17	0.02 to 0.33
Educational level_primary	0.007	-0.17 to 0.18	-0.03	-0.19 to 0.13
Educational level_secondary and above	0.44	0.24 to 0.63	0.17	**0.14 to 0.32** ^*^
Attitude toward CBHI_unfavourable	**Ref**			
Attitude toward CBHI_neutral	0.09	-0.08 to 0.26	-0.11	-0.24 to 0.01
Attitude toward CBHI_Favourable	0.73	0.57 to 0.89	0.31	**0.12 to 0.49** ^**^
Waiting time (in minutes)	-0.005	-0.006 to -0.004	-0.001	-0.002 to 0.000
CBHI status_Renwed				
CBHI status_Dropped	-1.07	-1.22 to—0.92	-0.23	**-0.41 to—0.05** ^*^
Distance from health facilities_> = 30 minutes	**Ref**			
Distance from health facilities_< 30 minutes	0.06	-0.11 to 0.23	-0.23	-0.23 to -0.03
Trust on constructed health facilities	0.36	0.29 to 0.44	0.05	-0.02 to 0.12
Wealth index_poorest	**Ref**			
Wealth index_Poor	0.09	-0.01 to 0.29	0.09	-0.07 to 0.24
Wealth index_Middle	-0.11	-0.31 to 0.08	0.05	-0.14 to 0.24
Wealth index_Rich	.006	-0.19 to 0.20	0.07	-0.09 to 0.23
Wealth index_Richest	0.12	-0.08 to 0.32	0.09	-0.06 to 0.24

**and *Denote statistical significance at the 1% and 5% level, respectively

Abbreviation: Ref = Reference group

This study showed that members’ satisfaction with what they experienced at health facilities and local CBHI offices had a positive association with trust in the CBHI scheme. Accordingly, for one unit increase in satisfaction score the trust score increased by 0.40 holding other covariates fixed [ß = 0.40 (95% CI: 0.41–0.57; p < 0.001)]. Another issue revealed by this study was that good perceived quality of services was positively affecting trust in the CBHI scheme. Accordingly, holding other covariates fixed, trust score increased by 0.15 among households who perceived good quality of services as compared to households who perceived poor quality of services[ß = 0.15 (95% CI: 0.02–0.29; p = 0.027)] (Table 3).

In addition, the attitude of CBHI members towards health insurance has a positive relationship with the trust of members in the health insurance scheme. Accordingly, keeping other variables constant, trust score increased by 0.31 among households who had a favorable attitude as compared to households who had an unfavorable attitude toward the CBHI scheme[ß = 0.31(95% CI: 0.12–0.49; p < 0.001]. Finally, a dropout from the CBHI scheme has a negative association with trust in the CBHI scheme. Accordingly, controlling other variables, households who discontinued their membership were found to have a statistically significant decrement of trust score by 0.23 as compared to households who renewed their membership [ß = 0.23 (95% CI: - 0.41–0.05; p = 0.013)] (Table 3).

## Discussion

The trust that potentially community-based health insurance (CBHI) members have in the actual health insurance scheme is a crucial determinant for whether or not community members decide to enroll and continue being members of the scheme. While the literature on informal health insurance in low-income countries mentions the importance of trust in consumers’ decision to insure today the association between trust and trust-building factors has not been researched in the Ethiopian context. Therefore, this study tried to examine factors affecting households’ trust in the CBHI scheme and a clear understanding of these factors plays a paramount role in developing structures and practices that build and sustain trust in the CBHI scheme.

This study showed that households with a secondary and above educational level increased their trust score as compared to households who were unable to read and write. Even though we didn’t find a study on relation of household heads’ educational status and trust in CBHI scheme, as best search of the author, a meta-analysis done in low and middle-income countries showed that a full range of quantitative studies supports the positive association between the education of the head of household and enrolment as well as willingness to renew their membership [17, 21]. The possible justification might be that educated people most probably had an understanding of the benefits packages, working principles and mechanisms of risk-sharing in health insurance; Hence, more likely to trust the CBHI scheme.

This study showed that members’ satisfaction with what they experienced at health facilities and the local CBHI office has a positive association with trust in the CBHI scheme. This finding is in line with a study done in Cambodia and Rwanda which also showed that individuals with poor previous experiences with other organizations were less willing to trust the CBHI scheme [18, 20]. The possible justification could be a failure to respond to members’ demands and patient affairs might impose a lack of trust in the CBHI scheme. However, increasing the availability and accessibility of service without compromising the quality irrespective of any criteria to fulfill members’ expectations will enhance the trust of members in the scheme so that they prefer to sustain being a member of the CBHI by renewing their membership.

This study revealed that dropout from the CBHI scheme has a negative association with trust in the CBHI scheme. Accordingly, controlling for other variables, households who discontinued their membership was found to have a statistically significant decrement of trust score as compared to household who renewed their membership. This finding was in line and contribute to what was already been found by a study conducted in Cambodia which also showed that households who had just enrolled, dropped out and never enrolled in CBHI had statistically significantly lower trust levels as compared to those who had renewed their membership [18]. In addition, another study revealed that for a one unit increase in trust score in the CBHI scheme the odds of dropout from CBHI decreased by 39% [17]. This finding insight that trust in the CBHI scheme is an important consideration in individuals’ decisions to continue being a member of the health insurance scheme or not.

This study showed that the attitude of CBHI members toward health insurance has a positive relationship with members’ trust in the health insurance scheme. Even though the author didn’t get a study that assessed the association between trust in the CBHI scheme and members’ attitude toward health insurance, one study conducted on Community-based health insurance and communities’ scheme requirement compliance in northeast Ethiopia showed that CBHI members who had a positive attitude toward CBHI management were more likely to comply with CBHI requirements than those members who had a negative attitude [35]. A possible reason for this could be that if members get promised services that meet their best interest which is sustainable they build trust in the scheme then stay being members. But, if the promised services are interrupted in between they lose their hope and suspect the scheme to see it in the future on other individuals other than being a member.

Our results observed that good perceived quality of services had a positive relationship with trust in the CBHI scheme. This finding is in line with a study conducted in Rwanda [36] and the possible reason for this could be members’ trust in the scheme benefits, the management, and health facilities that provide health services to the members. This finding indicates that the quality of services provided to the members plays a significant role in building trust as well as ensuring the sustainability of the scheme.

### Conclusion and recommendation

Our research examined factors affecting trust in the CBHI Scheme. Trust is an important factor for the CBHI scheme to enroll and maintain its members, given the amount of risk that is inherent in the nature of insurance schemes. This study found that the educational level of the household head, members’ satisfaction with what they experienced, members’ attitude toward CBHI, perceived quality of service and dropout from CBHI were covariates that had an association with trust in the CBHI scheme. Therefore, if the Manna district CBHI scheme wants to build trust and maintain membership great attention should be given toward the improvement of quality of service and attitude of members toward the CBHI scheme. In addition, we strongly recommend that the scheme should have to have concern and commitment to fulfill the best interests of the members which in turn increases members’ satisfaction so that they may have confidence to relay on and trust the scheme. These findings suggest that CBHI managers, different health insurance agencies, providers and policymakers need to think systematically about a wide range of initiatives that enhance trust and caring, and to design trust-building structures and practices that sustain the CBHI scheme.

### Strength and limitation of the study

As strength, the study was community based which enabled the generalizability of the study findings to the source population. In addition, this is the first study of its kind in the study area even at the country level and is believed to provide useful information for the existing CBHI scheme. As a limitation, this study might be prone to social desirability bias as an individual might respond to the question in a way it is acceptable to many other than telling their true feeling. This study also might be prone to recall bias as this study asks about the past 12 month illness history and health service utilization related issue.

## Supporting information

S1 Text Questionnaire used to assess factors affecting trust in CBHI scheme. (DOCX)

S1 Data Raw data used in the analysis of trust in CBHI Scheme. (SAV)
