# Supplementary material for: Factors affecting households’ trust in the community based health insurance scheme in Ethiopia
Source: PLOS Glob Public Health. 2022 May 4;2(5):e0000375. doi: 10.1371/journal.pgph.0000375 (PMC10022134; doi:10.1371/journal.pgph.0000375)
Supplement: S1 Text — (DOCX) [file pgph.0000375.s001.docx]

# Annex I: Questionnaires used to assess factors affecting households’ trust

**Section 1: Socio-economic and demographic characteristics of respondents**

Please ask the following question and write/circle the answer of participants

| No | Question | Response options | Code | Skip |
| --- | --- | --- | --- | --- |
| 101 | Age of the respondents | In years _________ |  |  |
| 102 | Residence | 1. Rural 2. Urban |  |  |
| 103 | Sex of the respondents | 1. Male 2. Female |  |  |
| 104 | Ethnicity | 1. Oromo 2. Amara  3. Tigre 4. Gurage  5. Other (Specify)________ |  |  |
| 105 | Religion | 1. Muslim 2. Protestant  3. Orthodox 4.Wakefata  5. Other (Specify)________ |  |  |
| 106 | Current marital status | 1. Married 2. Divorced  3. Separated 4. Widowed  5. Single |  |  |
| 107 | Number of an individual in the household | ___________in number |  |  |
| 109 | Are there under-five children in the household, how many? | ___________in number |  |  |
| 110 | What is your educational level? | 1. Unable to read and write  2. Only able to read and write  3. Primary education  4. Secondary education  5. Collage and above |  |  |
| 111 | What is your occupation? | 1. Farmer 2. Merchant  3. Daily laborer 4. Petty trader  5. Other(please specify_______) |  |  |

**Section II: CBHI information**

Please ask the following question and write/circle the answer of participants

| **No** | **Question** | **Response options** | **Code** | **Skip** |
| --- | --- | --- | --- | --- |
| 201 | CBHI Membership status | 1. Renewed 2. Dropped | If 2,=> | 204 |
| 202 | For how many times you have renewed your membership | __________ (times in No) |  |  |
| 203 | Do you have an intention to renew again when your current membership expires for next year | 1. Yes  2. No  3. I didn’t decide now |  |  |
| 204 | Do you have an intention to renew next round | 1. Yes  2. No  3. I didn’t decide now |  |  |
| 205 | After how many year of membership did you discontinue your membership | _________ (number of years) |  |  |

**A: Attitude of insured household towards CBHI**

Please ask the following statements and circle level of agreement of participants

1. Strongly disagree 2. Disagree 3. Neutral 4. Agree 5. Strongly agree

| No | **Item** | **Level of Agreement** | | | | |
| --- | --- | --- | --- | --- | --- | --- |
| 501 | CBHI has potential on promoting health care seeking behavior from modern health institutions | 1 | 2 | 3 | 4 | 5 |
| 502 | CBHI protects households from unaffordable healthcare expenditures. | 1 | 2 | 3 | 4 | 5 |
| 503 | Premium payment for the CBHI scheme is expensive. | 1 | 2 | 3 | 4 | 5 |
| 504 | CBHI is means of collecting revenue (profit) to the government. | 1 | 2 | 3 | 4 | 5 |
| 505 | CBHI scheme members receive a low quality of services than non-members. | 1 | 2 | 3 | 4 | 5 |
| 506 | Mistreatment of patients by professionals is common for members than non-members. | 1 | 2 | 3 | 4 | 5 |
| 507 | I did not have trust in the management and administration of the CBHI scheme. | 1 | 2 | 3 | 4 | 5 |
| 508 | CBHI is relevant only to promote the health condition of the poor. | 1 | 2 | 3 | 4 | 5 |
| 509 | Health insurance is good to pool the risk of health expenditures within the sick and the healthy. | 1 | 2 | 3 | 4 | 5 |
| 510 | CBHI should be advocated and scaled up to improve the health condition of a rural community. | 1 | 2 | 3 | 4 | 5 |
| TOTAL _____________ | | | | | | |

**Section IV: CBHI related factors**

**A: Affordability and expected returns from the insurance**

Please ask the following statements and circle level of agreement of participants

1. Strongly disagree 2. Disagree 3. Neutral 4. Agree 5. Strongly agree

| **No.** | **Question** | **Response options** | | | | |
| --- | --- | --- | --- | --- | --- | --- |
| 601 | The timing/time interval of premium payment is convenient for my household | 1 | 2 | 3 | 4 | 5 |
| 602 | The CBHI registration fee is easily affordable | 1 | 2 | 3 | 4 | 5 |
| 603 | The CBHI regular contribution (premium) is easily affordable | 1 | 2 | 3 | 4 | 5 |
| 604 | Received the promised benefit packages during membership | 1 | 2 | 3 | 4 | 5 |

**B: Experience of CBHI**

Please ask the following statements and circle level of agreement of participants

1. Strongly disagree 2. Disagree 3. Neutral 4. Agree 5. Strongly agree

| **No** | **Question** | **Response options** | | | | |
| --- | --- | --- | --- | --- | --- | --- |
| 701 | The local CBHI agent tries hard to solve CBHI implementation problems | 1 | 2 | 3 | 4 | 5 |
| 702 | The CBHI members have the right to guide and supervise the activities of the CBHI management | 1 | 2 | 3 | 4 | 5 |
| 703 | The CBHI benefit package meets the requirements of my household | 1 | 2 | 3 | 4 | 5 |
| 704 | I am satisfied with the experience at the local CBHI office when I go to register | 1 | 2 | 3 | 4 | 5 |
| 705 | I am satisfied with the local CBHI office when I go to pay the regular contribution (premium) | 1 | 2 | 3 | 4 | 5 |
| 706 | Members of CBHI is treated the same as non-members | 1 | 2 | 3 | 4 | 5 |
| 707 | Household members are satisfied with the information provided about CBHI | 1 | 2 | 3 | 4 | 5 |

**C: Trust of CBHI Members on schemes**

Please ask the following statements and circle level of agreement of participants

1. Strongly disagree 2. Disagree 3. Neutral 4. Agree 5. Strongly agree

| **No** | **Item** | **Level of Agreement** | | | | |
| --- | --- | --- | --- | --- | --- | --- |
| 801 | The community is involved in the management of the local CBHI scheme | 1 | 2 | 3 | 4 | 5 |
| 802 | Premium contributed by member used for CBHI purpose only | 1 | 2 | 3 | 4 | 5 |
| 803 | CBHI scheme is providing the reimbursement service | 1 | 2 | 3 | 4 | 5 |
| 804 | The local CBHI management is trustworthy | 1 | 2 | 3 | 4 | 5 |
| 805 | CBHI scheme distributes ID card as early as member enrolled/return as early as they send for renewal | 1 | 2 | 3 | 4 | 5 |

**Section V: Health service use related factors**

**A: Health status and health care-seeking behavior**

Please ask the following question and write/circle the answer of participants

| **No.** | **Question** | **Response options** | **Code** | **Skip** |
| --- | --- | --- | --- | --- |
| 901 | How do you rate the health status of you and your family? | 1. Very poor 2.Poor 3. Medium 4. Good 5. Very good |  |  |
| 902 | Does this household member suffer from a chronic disease? (symptoms for more than 30 days) | 1. 1. Yes 2. No |  |  |
| 903 | Frequency of recent illness episodes household within the past three months | __________ |  |  |
| 904 | Have you or any of your household members been ill during the last 12 months? | 1. 1. Yes 2. No |  |  |
| 905 | Does this household member seek medical treatment for the past 12 months from the nearest HF? | 1. 1. Yes 2. No |  |  |
| 906 | If yes, how many times did you go to the health facility for treatment in the past 12 months | __________ (in no.) |  |  |
| 907 | Generally, how would you perceive the quality of the health care service you had taken from HF (waiting time, medicine, diagnostics material)? Answer by thinking any facility that CBHI had a contractual agreement with in the past 12 months even if your answer for Q905 is no. | 1. Very poor 2. Poor  3. Medium 4. Good  5. Very good |  |  |
| 908 | What is your level of satisfaction with services you received from modern health care facilities after you become a member of CBHI? | 1. Very dissatisfied  2. Dissatisfied  3. Neither satisfied nor dissatisfied  4. Satisfied  5. Very satisfied |  |  |

**B: Access of health services related factors**

Please ask the following question and write/circle the answer

| **No** | **Question** | **Response options** | **Code** | **Skip** |
| --- | --- | --- | --- | --- |
| 1001 | What is the distance to the nearest health care facility from your home? | _________(in km) |  |  |
| 1002 | What mode of transport have you commonly used or would you use to travel to the nearest health care facility from your home? | 1. Walk 2. Bicycle  3. Motorbike 4. Car  5. Horse’s back  6. Other specify______ |  |  |
| 1003 | How many minutes do you take to walk to get the nearest public health facility from your home? | _________ (in minutes) |  |  |
| 1004 | Is the service time of public HF convenient? | 1. 1. Yes 2. No |  |  |
| 1005 | How much time do you and your family  Waited to get the services? | _________ (in minutes) |  |  |
| 1006 | Have you ever left a public HF without receiving treatment? | 1. Yes 2. No |  |  |
| 1007 | Has the public health facility you visited always had sufficient and necessary laboratory services? | 1. Yes 2. No |  |  |
| 1008 | Have you been obliged to purchase drugs outside the contracted health facility? | 1. Yes 2. No |  |  |
| 1009 | If yes, how would you describe the cost of purchasing these drugs? | 1. Very expensive  2. Expensive  3. Normal  4. Affordable  5. Very Affordable |  |  |

**C: Trust on contracted health facilities**

Please ask the following statements and circle level of agreement of participants

1. Strongly disagree 2. Disagree 3. Neutral 4. Agree 5. Strongly agree

| **No** | **Item** | **Level of Agreement** | | | | |
| --- | --- | --- | --- | --- | --- | --- |
| 1101 | health facility (HF) provides all services expected to be given at its level | 1 | 2 | 3 | 4 | 5 |
| 1102 | HF always has sufficient health professional | 1 | 2 | 3 | 4 | 5 |
| 1103 | HF always has sufficient drugs | 1 | 2 | 3 | 4 | 5 |
| 1104 | HF has improved referral system | 1 | 2 | 3 | 4 | 5 |
| 1105 | The physical facility is visually clean, attractive and comfortable | 1 | 2 | 3 | 4 | 5 |
| 1106 | HF staff has sufficient competency to treat a patient | 1 | 2 | 3 | 4 | 5 |
| 1107 | HF staff is committed in providing service | 1 | 2 | 3 | 4 | 5 |
| 1108 | HF provide services timely | 1 | 2 | 3 | 4 | 5 |
| 1109 | HF is concerned about the need for CBH member | 1 | 2 | 3 | 4 | 5 |
| 1110 | HF is reliable in handling the patient’s problems | 1 | 2 | 3 | 4 | 5 |

**D: Provider’s Attitude towards CBHI member**

Please ask the following statements and circle level of agreement of participants

1. Strongly disagree 2. Disagree 3. Neutral 4. Agree 5. Strongly agree

| **No** | **Item** | **Level of Agreement** | | | | |
| --- | --- | --- | --- | --- | --- | --- |
| 1201 | Health Professionals (HPs) well-come the clients in a good manner | 1 | 2 | 3 | 4 | 5 |
| 1202 | HPs provide enough time to insured patients | 1 | 2 | 3 | 4 | 5 |
| 1203 | HPs respond the right answer to all questions of clients | 1 | 2 | 3 | 4 | 5 |
| 1204 | HPs give equal treatment for members and nonmembers | 1 | 2 | 3 | 4 | 5 |
| 1205 | HPs never rude to a client during the consultation | 1 | 2 | 3 | 4 | 5 |
| 1206 | HPs treat a client with respect/courtesy | 1 | 2 | 3 | 4 | 5 |
| 1207 | HPs are always helpful for all patients | 1 | 2 | 3 | 4 | 5 |
| 1208 | HPs provide error-free services for CBHI members | 1 | 2 | 3 | 4 | 5 |
| 1209 | HPs have good discipline | 1 | 2 | 3 | 4 | 5 |
| 1210 | HPs respond immediately when called | 1 | 2 | 3 | 4 | 5 |

**E. Overall quality of service at a contracted health facility after CBHI is started**

Please ask the following statements and circle level of agreement

1. Increase 2. Decline 3. Similar 4. Do not know

| **No** | **Item** | **Response option** | | | |
| --- | --- | --- | --- | --- | --- |
| 1301 | Improvement of quality of overall healthcare services | 1 | 2 | 3 | 4 |
| 1302 | Improvements in the availability of the drug | 1 | 2 | 3 | 4 |
| 1303 | Availability of diagnostic/laboratory services | 1 | 2 | 3 | 4 |
| 1304 | Improvements in waiting time to get services | 1 | 2 | 3 | 4 |
| 1305 | Improvements in a referral system | 1 | 2 | 3 | 4 |
| 1306 | Improvements in the motivation of staff | 1 | 2 | 3 | 4 |
| 1307 | Improvements in the cleanliness of the health care institution | 1 | 2 | 3 | 4 |

**Section VI: Questions to assess the current family wealth index**

Ask the following and circle which households do have?

| **Asset type** |  | **Response** | | If yes please write a number |
| --- | --- | --- | --- | --- |
|  |  |  |  |  |
| **Domestic animals** |  |  |  |  |
|  |  |  |  |  |
| Ox | No |  | Yes |  |
|  |  |  |  |  |
| Cow | No |  | Yes |  |
|  |  |  |  |  |
| Calf | No |  | Yes |  |
|  |  |  |  |  |
| Sheep | No |  | Yes |  |
|  |  |  |  |  |
| Goat | No |  | Yes |  |
|  |  |  |  |  |
| Horse | No |  | Yes |  |
|  |  |  |  |  |
| Donkey | No |  | Yes |  |
|  |  |  |  |  |
| Cock and Hen | No |  | Yes |  |
|  |  |  |  |  |
| **Durable/non-productive assets** |  |  |  |  |
|  |  |  |  |  |
| Television | No |  | Yes |  |
|  |  |  |  |  |
| Radio | No |  | Yes |  |
|  |  |  |  |  |
| Electricity | No |  | Yes |  |
|  |  |  |  |  |
| Refrigerator | No |  | Yes |  |
|  |  |  |  |  |
| Conventional telephone | No |  | Yes |  |
|  |  |  |  |  |
| Mobile phone | No |  | Yes |  |
|  |  |  |  |  |
| Motorcycle | No |  | Yes |  |
|  |  |  |  |  |
| Cycle | No |  | Yes |  |
| Cart | No |  | Yes |  |
|  |  |  |  |  |
| Gold | No |  | Yes | In gram______ |
|  |  |  |  |  |
| Ownership of an owned living house | No |  | Yes |  |
|  |  |  |  |  |
| Ownership of agricultural land | No |  | Yes | In hector______ |
|  |  |  |  |  |
| **Productive assets** |  |  |  |  |
|  |  |  |  |  |
| Plough | No |  | Yes |  |
|  |  |  |  |  |
| Axe | No |  | Yes |  |
|  |  |  |  |  |
| Hoe | No |  | Yes |  |
|  |  |  |  |  |
| Shovel | No |  | Yes |  |
|  |  |  |  |  |
| Sickle | No |  | Yes |  |
|  |  |  |  |  |
| Modern beehive | No |  | Yes |  |
|  |  |  |  |  |
| Traditional beehive | No |  | Yes |  |
|  |  |  |  |  |
| **Housing utilities** |  |  |  |  |
|  |  |  |  |  |
| Indoor plumping/ pipe water | No |  | Yes |  |
|  |  |  |  |  |
| Type of flooring | Earth/dung |  | Cement/raw wood |  |
|  |  |  |  |  |
| Toilet facility (please observe) | Traditional toilet |  | Improved latrine |  |
|  |  |  |  |  |
| **Other household materials** |  |  |  |  |
| Sofa | No |  | Yes |  |
|  |  |  |  |  |
| Bed | No |  | Yes |  |
|  |  |  |  |  |
| Table | No |  | Yes |  |
|  |  |  |  |  |
| Chair | No |  | Yes |  |
| Stove | No |  | Yes |  |
| Vehicle | No |  | Yes |  |
|  |  |  |  |  |

**Kutaa I: Gaaffii Odeeffannoo Walii Galaa maati**

Gaaffile armaan gadii gaafadhuu deebi isaa barreessi ykn ittii marii

| **T.L** | **Gaaffiilee** | **Deebii** | **Yaadachisa** | **darbinsa** |
| --- | --- | --- | --- | --- |
| 101 | Umuriin keessan meeqaa? | Umurii waggaan_________ |  |  |
| 102 | Bakka jireenya keessanii eessaa? | 1. Baadiyyaa 2. Magaala |  |  |
| 103 | Saala | 1. Dhiira 2. dhalaa |  |  |
| 104 | Sabummaan keessan maalii? | 1. Oromoo 2. Amaharaa  3. Tigree 4. Guraage  5. kan biroo (ibsii)________ |  |  |
| 105 | Amantaan keessan maalii? | 1. Muslimaa  2. Protestantii  3. Orthodoxii 4.Waaqeffataa  5.kan biroo(ibsii)________ |  |  |
| 106 | Haala fuudha fi heerumaa keessani akkaamii? | 1. Kan fuudhe ykn heerumte 2. Kan irraa du’e  3. Kan hiike /hiikte  4. Kan addaan ba’e /baate  5. Kan hin fuudhin ykn heerumiin |  |  |
| 107 | Lakkoofsa maatii mana kana keessan keessa jiraatanii meeqa? | Lakkoofsaan ___________ |  |  |
| 108 | Baay’inni namoota umurii 64 olii maati keessan keessa meeqaa? | Lakkoofsaan ___________ |  |  |
| 109 | Baay’inni daa’immanii umurii 0-5 jiranii maati kana keessa meeqa? | Lakkoofsaan ___________ |  |  |
| 110 | Sadarkaa barnootaa keessani maalii? | 1. Dubbisuuf barressuu kan hin dandeenye  2. Dubbisuuf barressuu kan danda’uu  3. Sadarkaa tokkoffaa  4. Sadarkaa lammaffaa  4. Collejji fi isaa ol |  |  |
| 111 | Hojiin keessan maalii? | 1. Qotee bulaa 2. Dandalaa  3. Dafqaan bulaa 4. Dadalaa xixiqqaa daandii qarqaraa  5. Kan biro( ibsii) |  |  |

**Kutaa II: Odeeffanno waliigalaa dhimma Insuraansii Fayyaa Hawaasa (IFH)**

Gaaffile armaan gadii gaafadhuu deebi isaa barreessi ykn ittii marii

| 201 | Haala miseensumma kessani maalii? | 1. Kan haareffate  2.Addan kan kute | 2, => | 204 |
| --- | --- | --- | --- | --- |
| 202 | Marsaa meeqaffaf miseensummaa keessan hareffattan? | ________( lakk) |  |  |
| 203 | Kan bara dhufuu haareffachuuf yaaddani jirtu? | 1. Eeyyee  2. Lakki  3. Ammaaf hin murtessine |  |  |
| 204 | Marsaa isa dhufutti haareffachuuf qophii qabdaa? | 1.Eeyyee  2.Lakki  3. Ammaaf hin murtessine |  |  |
| 205 | Wagga meeqa ergaa fayyadamtanii booda miseensumma IFH keessan addaan kuttan? | ___________ lakk |  |  |

**Kutaa III: Sababoota dhuunfa ykn maatii miseensa wallin wal qabatan**

**A: Haala hirmaanna IFH fi Miseensumma waldaa hawaasaa:**

Gaaffile armaan gadii gaafadhuu lakkofsa deebii deebi isaa ittii marii

| **T.L** | **Gaaffilee** | **Filannoo deebii** | **yadachiisa** | **darbinsaa** |
| --- | --- | --- | --- | --- |
| 401 | Maatii kana keessa namni leenjii ykn walgahii ykn odeeffanoo dhimma IFH kennamu irratti hirmaate beeku jiraa? | 1.Eeyyee 2.Lakki |  |  |
| 402 | Yoo jiraatee si’a meeqa hirmaatee? | _______(lakk) |  |  |
| 403 | Isin ykn maati keessan keessa namnii dhimma to’anno IFH irratti hirmaate beeku jira? | 1.Eeyyee 2.Lakki |  |  |
| 404 | Maati kana keessa namni bakka gaggessuma (official position) qabatee beeku jiraa? | 1.Eeyyee 2.Lakki |  |  |
| 405 | Miseensa waldaa hawaasaa keessatti hirmaattanii beektuu? | 1.Eeyyee 2.Lakki |  |  |
| 406 | Yoo eeyyee ta’ee waldaa hawaasaa kam keessa jirtuu/hirmaattanii beektuu | 1. Daboo  2. Uquubii  3.Afoosha/iddiri  4. Liqii fi qusannaa  5. Waldaa karaa amantii |  |  |

**B: Ilaalcha miseensaa IFH irratti**

Gaaffile armaan gadii gaafadhuu sadarkaa waligaitee deebii gaaffii ittii marii

1. Baayyeen irratti walii hin galu 2.Irratti walii hin galu 3 Giddugaleessa 4.Irrattiin walii gala 5. Baayyeen irratti walii gala

| **T.L** | **Gosa gaaffilee** | **Sadarkaa walii galtee** | | | | |
| --- | --- | --- | --- | --- | --- | --- |
| **501** | IFH dhabbata fayya ammayawa irra amala tajaajila fayya argachuu barbaadu jajjabeessuf dandeetti qaba. | 1 | 2 | 3 | 4 | 5 |
| **502** | IFH baasii tajajila fayya kaffalamuuf hin danda’amnerra maatii in eega | 1 | 2 | 3 | 4 | 5 |
| 503 | Kaffaltiin duraa IFH’f kaffalamu jabeenya | 1 | 2 | 3 | 4 | 5 |
| 504 | IFH jechuun karaa ittiin galiin bu’adhaaf mootummaaf walitti qabuudha | 1 | 2 | 3 | 4 | 5 |
| 505 | Miseensi IFH tajaajila qulqullinni isaa waarra miseensa hin taane irraa gadi bu’aa ta’ee argata | 1 | 2 | 3 | 4 | 5 |
| 506 | Yaaliin dogongoraa ogeessaan raawatamu warra miseensa hin taannerra warra miseensa ta’an irratti yeroo baay’ee mul’ata | 1 | 2 | 3 | 4 | 5 |
| 507 | To’annoo fi bulchiinsa dame IFH irratti amantaa hin qabu | 1 | 2 | 3 | 4 | 5 |
| 508 | IFH haala fayya warra hiyyessa qofaa fooyyessuuf barbaachisa | 1 | 2 | 3 | 4 | 5 |
| **509** | IFH baasii balaa fayyaatif ooluu nama isa dhukkubsata fi faayya giddutti qooduf gaari dha. | 1 | 2 | 3 | 4 | 5 |
| **510** | IFH haala fayyaa hawaasa fooyyessuuf jajjabeeffamu akkasumas babal’achuu qaba | 1 | 2 | 3 | 4 | 5 |
| **IDA’AMA ___________** | | | | | | |

**Kutaa IV: Dhimmoota dhaabbata insuraansi waliin wal qabatan**

**A: Haala kaffaltii fi faayidaa IFH irraa**

Gaaffile armaan gadii gaafadhuu sadarkaa waligaitee deebii ykn lakkoofsa deebii gaaffii ittii marii

1. Baayyeen irratti walii hin galu 2.Irratti walii hin galu 3 Giddugaleessa 4.Irrattiin walii gala 5. Baayyeen irratti walii gala

| **T.L** | **Gaaffilee** | **Sadarka waligalinsa** | | | | |
| --- | --- | --- | --- | --- | --- | --- |
| 601 | Yeroon kaffaltii/yeroon turtii kaffaltii buusi maatii koof mijatadha | 1 | 2 | 3 | 4 | 5 |
| 602 | Kaffaltiin galmee IFH salphatti kaffalama | 1 | 2 | 3 | 4 | 5 |
| 603 | Kafaltiin buusi inshuraansii fayyaa salphatti kaffalama | 1 | 2 | 3 | 4 | 5 |
| 604 | Tajaajila fayya akkan argattuuf waada galame yeroo turtii miseensumma keessatti argatteta. | 1 | 2 | 3 | 4 | 5 |

**B: Gaaffilee Muuxanno fi haala Caaseffama dhaabbata IFH**

Gaaffile armaan gadii gaafadhuu sadarkaa waligaitee deebii gaaffii ittii marii

1. Baayyeen irratti walii hin galu 2.Irratti walii hin galu 3.Giddugaleessa 4.Irrattiin walii gala 5. Baayyeen irratti walii gala

| **T.L** | **Gaaffilee** | **Sadarkaa irratti waligalinsa** | | | | |
| --- | --- | --- | --- | --- | --- | --- |
| 701 | Gaggeessitonni IFH naanno jiran rakko hojiirra oolma IFH furuuf sirritti jabaatanii hojjecha jiruu. | 1 | 2 | 3 | 4 | 5 |
| 702 | Miseensi IFH hojii to’anno IFH keessatti kallatti agarsiisu akkasumas hordofuuf mirga qaba. | 1 | 2 | 3 | 4 | 5 |
| 703 | Tajaajilli fayyaa IFH jalatti hammatame fedhii maati koo guutera | 1 | 2 | 3 | 4 | 5 |
| 704 | Yeroon galmeef deeme muuxanno wajjira IFH naanno jirutti quufera | 1 | 2 | 3 | 4 | 5 |
| 705 | Yeroon buusi dhaabbata kiyya kaffaluuf deemuu waajjira IFH naanno jirutti quufera | 1 | 2 | 3 | 4 | 5 |
| 706 | Miseensi IFH akkuma warra kiisidha baase yaalamutti (warra miseensa hin taane) yaala argata | 1 | 2 | 3 | 4 | 5 |
| 707 | Miseensii odeeffanno dhimma IFH irratti kenname irratti itti quufinsa qabu | 1 | 2 | 3 | 4 | 5 |

**C: gaaffilee amantaa miseensi IFH dhaabbata IFH irratti qabuu**

Gaaffile armaan gadii gaafadhuu sadarkaa waligaitee deebii gaaffii ittii marii

1. Baayyeen irratti walii hin galu 2.Irratti walii hin galu 3.Giddugaleessa 4.Irrattiin walii gala 5. Baayyeen irratti walii gala

| **T.L** | **Gosa gaaffilee** | **Sadarkaa waligalinsa** | | | | |
| --- | --- | --- | --- | --- | --- | --- |
| 801 | Hawaasni hojii to’anno dhaabbata IFH naanno jiruu keessatti ni hirmaata | 1 | 2 | 3 | 4 | 5 |
| 802 | Buusin miseensarra walitti qabamee dhimma hojii IFH qofaaf oola | 1 | 2 | 3 | 4 | 5 |
| 803 | Dhaabbatni IFH tajaajila baasi bakka bu’insaa miseensaf ni kenna | 1 | 2 | 3 | 4 | 5 |
| 804 | Hooggansi IFH naanno amansiisadhaa | 1 | 2 | 3 | 4 | 5 |
| 805 | Waraqaa eenyumma akkuma galmoofneen yeroon nuuf kennamera/yeroo haareffachuuf ergine dafee nuuf deebi’eera | 1 | 2 | 3 | 4 | 5 |

**Kutaa V: Dhimmoota fayyaa fi itti fayyadama dhaabata fayya waliin wal qabatan**

Gaaffile armaan gadii gaaffadhuu lakkoofsa deebii itti marii;

**A: Haala fayyaa fi amala fayyadamummaa tajaajila fayyaa**

| **T.L** | **Gaaffilee** | **Filannoo debii** | **koodii** | **darbinsa** |
| --- | --- | --- | --- | --- |
| 901 | Walumaagalatti haala fayyaa kee fi maatii kanaa akkamiin ibsita? | 1. Baayyee badaa dha  2. Badaa dha  3. Giddu galeessa  4. Gaarii dha  5. Baayyee gaarii dha |  |  |
| 902 | Maati kana keessa namni dhukkuba yeroo dheeran rakkatu jiraa (mallatto guyyaa dhukkubaa guyyaa 30 oliif ture yeroo qoranno kanaatti) | 1.eeyyee 2.lakki |  |  |
| 903 | ji’oota sadandaraban keessatti maatii kana kessatti dhukkubni si’aa meeqa deddebi’ee | ________(lakk) |  |  |
| 904 | Ji’oota 12’n darban kana keessatti maatii kana keessa namni dhukkuba yeroo gabaaba, dhukkuba akka tasaa ykn miidhamni irra gahee jiraa | 1.eeyyee 2.lakki |  |  |
| 905 | Ji’oota 12 darban keessatti mana yaala deemtee beektaa? | 1.eeyyee 2.lakki |  |  |
| 906 | Yoo eeyyee ta’ee yeroo meeqa yaala argachuuf demtee? | __________ (lakk) |  |  |
| 907 | Walumaa galatti haala qulqullina tajaajila fayyaa siif kenname akkami jette fudhatta?(yoo deebiin gaaffi 905 lakki ta’ee dhaabbata fayyaa kan biraa itti tajaajilmte yaaduun deebisi) | 1. Baay’ee gadi aanaa  2. Gadi aanaa  3. Giddugaleessa  4. Olaanaa  5. Baay’ee olaanaa |  |  |
| 908 | Erga miseensa IFH taatee tajaajila dhaabbata fayyaa ammayyaawa irraa argatte irratti sadarkaan itti quufinsi kee maal fakkaataa | 1. Baay’ee Itti hin quufne  2. Itti hin quufne  3.Giddu galeessa  4. Itti quufera  5. Baay’ee itti quufera |  |  |

**B: Dhiheenya fi argamsa tajaajila fayyaa;**

**Gaaffilee armaan gadii gaafadhuu lakkofsa deebi itti marii**

| **T.L** | **Gaaffilee** | **Filannoo deebii** | **koodi** | **darbinsa** |
| --- | --- | --- | --- | --- |
| 1001 | Fageenyi mana kee fi dhaabbata fayyaa dhiheenya kee jiru hagamii | _________(km) |  |  |
| 1002 | Mana irra gara dhaabbata fayyaa dhiheenya kee jiruu akkamiin deemtaa? | 1. Miilan 2.Saayikilii  3. Motorsaayikilii  4. Konkolaata  5. Fardaan  6.Kanbiroo(ibsii___) |  |  |
| 1003 | Mana keerra gara dhaabbat fayyaa miilan deemun hagam sitti fudhattaa? | ______ (daqiiqa) |  |  |
| 1004 | Yeroon tajaajilaa dhaabbata fayyaa siif mijataadha | 1. Eeyyee  2. Lakki |  |  |
| 1005 | Atiif maatin kee dhaabbata fayyaa deemtee tajaajila argachuuf yeroo hagamii turtaa | _____ (daqiiqa) |  |  |
| 1006 | Osoo tajaajila yaalaa argachuuf deemtee hin argatiin dhaabbata fayya keessa baate beekta? | 1. Eeyyee  2. Lakki |  |  |
| 1007 | Dhaabbati fayyaa yeroo maraa tajaajila laboratoorii barbaachisaa fi gahaa ta’e ni qabaa | 1. Eeyyee  2. Lakki |  |  |
| 1008 | Yaalaf deemtee qorichaa dhabbata fayyaa alaatii bituuf dirqamtee beektaa? | 1. Eeyyee  2. Lakki |  |  |
| 1009 | Yoo eeyyee tahee gatii qoricha alaa bittee akkamitti ibsitaa? | 1. Baay’ee mi’aadha  2. Mi’aadha  3. Giddu galeessa  4. Ni bitamaa  5. Salphatti bitama |  |  |

**C: Amantaa miseensaa dhaabbata fayya walii galtee uumee irratti qabu**

Gaaffile armaan gadii gaafadhuu sadarkaa waligaitee deebii gaaffii ittii marii

1. Baayyeen irratti walii hin galu 2.Irratti walii hin galu 3 Giddugaleessa 4.Irrattiin walii gala 5. Baayyeen irratti walii gala

| **T.L** | **Gosa gaaffilee** | **Sadarkaa walii galinsaa** | | | | |
| --- | --- | --- | --- | --- | --- | --- |
| 1101 | Dhaabbati fayyaa tajaajila sadarkaa isaatti kennu qabuu hundaa kennaa jiraa | 1 | 2 | 3 | 4 | 5 |
| 1102 | Dhaabbati fayyaa yeroo hundaa ogeessa gahaa ni qabaa | 1 | 2 | 3 | 4 | 5 |
| 1103 | Dhaabbati fayyaa yeroo maraa qoricha barbaachisaa ta’e ni qabaa | 1 | 2 | 3 | 4 | 5 |
| 1104 | Dhaabbati fayyaa sirna walharkaa fuudhinsa dhukkubsata fooyya’aa ta’ee ni qabaa | 1 | 2 | 3 | 4 | 5 |
| 1105 | Dhaabbati fayyaa yeroo ijaan ilaalamu naannawaan is qulqulluu, hawwata fi mijataa dha | 1 | 2 | 3 | 4 | 5 |
| 1106 | Ogeessi dhaabbata fayya dhukkubsata yaaluuf gahumsa ga’aa qabuu | 1 | 2 | 3 | 4 | 5 |
| 1107 | Ogeessi dhaabbata fayya dhukkubsata yaaluuf ni dhimmamu | 1 | 2 | 3 | 4 | 5 |
| 1108 | dhaabbati fayyaa tajaajila yeroodhan laata | 1 | 2 | 3 | 4 | 5 |
| 1109 | Dhaabbati fayyaa fedhii miseensa IFH guutuf ni dhimma | 1 | 2 | 3 | 4 | 5 |
| 1110 | Dhaabbati fayya rakko dhukkubsata furuuf ni amanama | 1 | 2 | 3 | 4 | 5 |

**D: Gaaffilee haala miseensi IFH ilaalcha ogeessa fayyaa irratti**

Gaaffile armaan gadii gaafadhuu sadarkaa waligaitee deebii gaaffii ittii marii

1. Baayyeen irratti walii hin galu 2.Irratti walii hin galu 3 Giddugaleessa 4.Irrattiin walii gala 5. Baayyeen irratti walii gala

| **T.L** | **Gosa gaaffiilee** | **Sadarkaa irrattin waligalinsa** | | | | |
| --- | --- | --- | --- | --- | --- | --- |
| 1201 | Ogeessi fayya dhukkubsata haala gaarin simataa | 1 | 2 | 3 | 4 | 5 |
| 1202 | Ogeessi fayyaa dhukkubsattotaaf yeroo gahaa ni kenna | 1 | 2 | 3 | 4 | 5 |
| 1203 | Ogeessi fayyaa gaaffi dhukkubsataa hundaaf deebi sirri ni kenna | 1 | 2 | 3 | 4 | 5 |
| 1204 | Ogessi fayyaa tajaajila yaalaa miseensa fi mit-miseensaaf wal qixa kenna | 1 | 2 | 3 | 4 | 5 |
| 1205 | Ogeessi fayya yeroo qoranno tasuma dhukkubsata hin dheekkamu | 1 | 2 | 3 | 4 | 5 |
| 1206 | Ogessi fayyaa dhukkubsata bifa kabajaa qabuun yaala | 1 | 2 | 3 | 4 | 5 |
| 1207 | Ogessi fayyaa yeroo hundaa dhukkubsata maraaf amala nama gargaaru kan qabanidha | 1 | 2 | 3 | 4 | 5 |
| 1208 | Ogeessi tajajaajila dogoggora irraa walaba ta’e miseensa IFH ni kenna | 1 | 2 | 3 | 4 | 5 |
| 1209 | Ogeessi fayyaa amala gaarii qabu | 1 | 2 | 3 | 4 | 5 |
| 1210 | Ogeess fayya yeroo waamaman hatattamaan deebi laatu | 1 | 2 | 3 | 4 | 5 |

**E.Fooyya’insa qulqullina tajaajila fayya;**

Gaaffile armaan gadii gaafadhuu sadarkaa waligaltee deebii gaaffii ittii marii

1. Dabaleera, 2.Hira’ateera 3.Wal hin caaluu, 4. Hin beeku

| **No** | **Item** | **Fiannoolee deebii** | | | |
| --- | --- | --- | --- | --- | --- |
| 1301 | Haalli walii gala qulqullina tajaajial fayyaa | 1 | 2 | 3 | 4 |
| 1302 | Fayyai’nsa argama qorichaa irratti | 1 | 2 | 3 | 4 |
| 1303 | Foyya’insa argama tajaajila qorannoo/laboratory | 1 | 2 | 3 | 4 |
| 1304 | Fooya’insa yeroo turtii tajaajila argachuu | 1 | 2 | 3 | 4 |
| 1305 | Fooyya’insa haala ol-erginsa dhukkubsataa | 1 | 2 | 3 | 4 |
| 1306 | Fooyya’insa kaka’umsa ogeessa fayyaa | 1 | 2 | 3 | 4 |
| 1307 | Fooyya’insa haala qulqullina moora dhaabbat fayyaa | 1 | 2 | 3 | 4 |

**Kutaa VI; Gaaffiilee Haala Qabeenya Maatii Ilaallatan**

Kanneen armaan gadii keessaa kan isaan qabaan gafadhuu lakkofsa deebi barreessi?

| **Gosa qabeenyaa** | **Deebii** | | **Yoo 2 ta’ee baay’na isaa barreessii** |
| --- | --- | --- | --- |
| **Beeylada** | 1.Lakkii | 2.Eeyyee |  |
| sangaa | 1.Lakkii | 2.Eeyyee |  |
| Sa’aa | 1.Lakkii | 2.Eeyyee |  |
| Jabbii | 1.Lakkii | 2.Eeyyee |  |
| Hoolaa | 1.Lakkii | 2.Eeyyee |  |
| Re’ee | 1.Lakkii | 2.Eeyyee |  |
| Fardaa | 1.Lakkii | 2.Eeyyee |  |
| Harree | 1.Lakkii | 2.Eeyyee |  |
| lukkuu | 1.Lakkii | 2.Eeyyee |  |
| **Meeshaalee dhaabbataa** |  |  |  |
| Televizini | 1.Lakkii | 2.Eeyyee |  |
| Raadiyoo | 1.Lakkii | 2.Eeyyee |  |
| Elektrikii | 1.Lakkii | 2.Eeyyee |  |
| Firijii | 1.Lakkii | 2.Eeyyee |  |
| Bilbila manaa | 1.Lakkii | 2.Eeyyee |  |
| Bilbila moobayilii | 1.Lakkii | 2.Eeyyee |  |
| motorsaayikilii | 1.Lakkii | 2.Eeyyee |  |
| Saayikilii | 1.Lakkii | 2.Eeyyee |  |
| Gaarii | 1.Lakkii | 2.Eeyyee |  |
| Faaya (warqee) | 1.Lakkii | 2.Eeyyee | Gramiin __ |
| Mana jireenya dhuunfaa | 1.Lakkii | 2.Eeyyee |  |
| Lafa qotisaa dhuunfa | 1.Lakkii | 2.Eeyyee | Hektaaran _ |
| **Meeshalee oomishaa** |  |  |  |
| Maarashaa | 1.Lakkii | 2.Eeyyee |  |
| Qottoo | 1.Lakkii | 2.Eeyyee |  |
| Doomaa | 1.Lakkii | 2.Eeyyee |  |
| Akaafaa | 1.Lakkii | 2.Eeyyee |  |
| Haamtuu | 1.Lakkii | 2.Eeyyee |  |
| Gaagura ammayyaa | 1.Lakkii | 2.Eeyyee |  |
| Gaagura aadaa | 1.Lakkii | 2.Eeyyee |  |
| **Haala mana jireenya** |  |  |  |
| Bishaan ujummoo | 1.Lakkii | 2.Eeyyee |  |
| Lafa mana keessa | 1.Biyyee | 2.Cimintoo/xaawulaa |  |
| Haala mana fincaanii | 1.Qulqullina kan kan hin qabne | 2.Kan qulqullina qabu |  |
| **Qabeenya manaa kan biroo** |  |  |  |
| Soofaa | 1.Lakkii | 2.Eeyyee |  |
| Siree ciisichaa | 1.Lakkii | 2.Eeyyee |  |
| Xarapheezza | 1.Lakkii | 2.Eeyyee |  |
| Taa’umsa | 1.Lakkii | 2.Eeyyee |  |
| Istoovii | 1.Lakkii | 2.Eeyyee |  |
| konkolaataa | 1.Lakkii | 2.Eeyyee |  |
